# Imported dengue in Buenos Aires, Argentina.

**DOI:** 10.3201/eid0606.000619

**Published:** 2000

**Authors:** A Seijo, D Curcio, G Avilés, B Cernigoi, B Deodato, S Lloveras


when aggressive male animals fight. Although this pattern has been observed for several host species of New World hantaviruses, this is the first known demonstration for Dobrava virus and *A. flavicollis*. Of the two female rodents with evidence of hantavirus infection, one had scars, and one did not. The latter was positive by PCR on lung tissue but did not have detectable antibody in blood, which perhaps indicates very recent infection.

The seropositive *R. rattus* from Pramanta is the first evidence of hantavirus infection in *Rattus* within Greece. No *Rattus* captured during previous expeditions had hantavirus antibody (8). The low antibody titer (1:32) and failure to amplify viral RNA by PCR from this animal could indicate infection with a heterologous hantavirus with low cross-reactivity. Perhaps more likely, the antibody detected in this 39-g juvenile rat may represent waning maternal antibody. Transfer of protective maternal antibody to *R. norvegicus* pups by Seoul virus-infected dams has been demonstrated (9).

Our data implicate *A. flavicollis* as the reservoir of Dobrava virus in northern Greece and demonstrate the common occurrence of that species in both sylvatic and peridomestic habitats. These preliminary results underscore the need for continued, more intensive reservoir studies in Greece.

### Acknowledgments

We gratefully acknowledge Jon Dunnum for helping identify small mammal specimens and Stuart Nichol for helping in the phylogenetic analysis.

This study was supported by the World Health Organization, grant no. A18/286/2GRE.

**Anna Papa,* James N. Mills,† Sophie Kouidou,* Benjiang Ma,* Evagelia Papadimitriou, and Antonis Antoniadis***
*Aristotelian University of Thessaloniki, Thessaloniki, Greece; and †Centers for Disease Control and Prevention, Atlanta, Georgia, USA

### References

1. Antoniadis A, Stylianakis A, Papa A, Alexiou-Daniel S, Lambropoulos A, Nichol ST, et al. Direct genetic detection of Dobrava virus in Greek and Albanian haemorragic fever with renal syndrome (HFRS) patients. J Infect Dis 1996;174:407-10.
2. Papa A, Johnson AM, Stockton PC, Bowen MD, Spiropoulou CF, Ksiazek TG, et al. Retrospective genetic study of the distribution of hantaviruses in Greece. J Med Virol 1998;55:321-7.
3. Avsic-Zupanc T. Hantaviruses and hemorrhagic fever with renal syndrome in the Balkans. In: Saluzzo JF, Dodet B. Factors in the emergence and control of rodent-borne viral diseases. Amsterdam: Elsevier;1999: p. 93-8.
4. Papa A, Spiropoulou C, Nichol S, Antoniadis A. Tracing Dobrava hantavirus infection. J Infect Dis 2000;181:2116-7.
5. Nemirov K, Vapalahti O, Lundkvist Å, Vasilenko V, Golovljova I, Plyusnina A, et al. Isolation and characterisation of Dobrava hantavirus carried by the striped field mouse (*Apodemus agrarius*) in Estonia. J Gen Virol 1999;80:371-9.
6. Mills JN, Childs JE, Ksiazek TG, Peters CJ, Velleca WM. Methods for trapping and sampling small mammals for virologic testing. Atlanta: U.S. Department of Health and Human Services;1995.
7. Glass GE, Childs JE, Korch GW, LeDuc JW. Association of intraspecific wounding with hantaviral infection in wild rats (*Rattus norvegicus*). Epidemiol Infect 1988;101:459-72.
8. LeDuc JW, Antoniades A, Siamopoulos K. Epidemiological investigations following an outbreak of hemorrhagic fever with renal syndrome in Greece. Am J Trop Med Hyg 1986;35:654-9.
9. Dohmae K, Nishimune Y. Protection against hantavirus infection by dam's immunity transferred vertically to neonates. Arch Virol 1995;140:165-72.

### Imported Dengue in Buenos Aires, Argentina

**To the Editor:** After more than 70 years without reports of cases, an outbreak of dengue (type 2) occurred in the northwestern region of Argentina from January to May 1998; 818 cases of denguelike illness were reported (incidence rate: 45/10,000 inhabitants) (1). The outbreak was restricted to a few cities of the Chaco Salteño Region.

The last dengue epidemic in Argentina (in 1926) (2) affected the Mesopotamia Region and Rosario City. An earlier widely distributed epidemic in 1916 occurred in the coastal region along the Uruguay River (Corrientes and Entre Ríos provinces), reached Parana City (along the Parana River), and affected approximately 50% of the city's population (3). Both outbreaks began in Paraguay. No cases were detected in Buenos Aires.

High numbers of *Aedes aegypti* are reported in all places where surveillance for these vectors is conducted in Argentina. The Breteau rate (a measure of vector density; the number of positive containers is divided by the number of inspected houses) in the Federal District averaged >40% in the first trimester of 2000 and was 30% to 80% in suburban districts in 1999 (R. Boffi, Ministerio de Salud de la Nación; N. Schweigmann, University of Buenos Aires, pers. commun.).

In Argentina's neighboring countries, dengue is a serious public health problem. From December 1999 through March 2000, Paraguay reported 42,000 dengue cases, 9 of dengue hemorrhagic fever (4). Brazil has reported cases of dengue and dengue hemorrhagic fever, and Bolivia has reported dengue and a large yellow fever outbreak (4). From December 1999 to March 2000, 85 patients with denguelike illness arrived in Buenos Aires from one of these countries' dengue-epidemic areas and were seen at F. J. Muñiz Hospital in Buenos Aires. An enzyme-linked immunosorbent assay-capture immunoglobulin M test (commercial kit) (5) and a plaque reduction neutralization test on cell culture were performed (6). Laboratory diagnosis of dengue infection was made in 38 cases. Twenty-five cases were in female patients, and 13 were in male patients; the age range of patients was 8 to 74 years (average, 39 years). All patients were Argentinean residents; 18 (47.4%) lived in the Federal District, and 20 (52.7%) in the suburban area (west and south). Except for one patient who had traveled to Saint Thomas Island, the patients traveled from Paraguay (Asunción, Ciudad del Este, Luque, and other cities). The patients had been out of Argentina 4 to 60 days (average, 17 days). Twenty-six (68.4%) patients had viremia in their place of residence (Federal District or suburbs). In Buenos Aires, 20 patients had viremia for 5 days, 3 patients for 4 days, and 3 patients for 3 days. Ten patients (26.3%) had mild febrile illness; 23 (57.1%) had classic dengue fever; and 5 (13.2%) had dengue fever with hemorrhage. Four patients had epistaxis, and one woman had self-limited, abnormal vaginal bleeding of 24 hours' duration.

Considering *A. aegypti* infestation rates and the large population of this area, (3 million in the Federal District and 8 million in the suburban areas) (7), the probability of an outbreak is high. Historically, the highest rates for *A. aegypti* in this area are reported in April and May (8). In 1997, 1,608,062 tourists arrived from countries that have dengue transmission (1,135,168 from neighboring countries, 358,286 from Paraguay) (9). Approximately 40% of these tourists arrived by plane. In 1998, >700,000 Argentineans left the country through Buenos Aires to travel to countries where dengue transmission occurs (7). Migration through bordering areas, especially in tropical regions of northern Argentina, is underreported.

The number of imported dengue cases in Buenos Aires and other cities in Argentina detected in the current period is substantially higher than the number detected in previous years. Argentina is at risk for an outbreak of dengue, and the health system of the country should be preparing for it.

**Alfredo Seijo,* Daniel Curcio,* Gabriela Avilés,† Beatriz Cernigoi,* Bettina Deodato,* and Susana Lloveras***

*Hospital de Infecciosas F.J. Muniz, Buenos Aires, Argentina; †Instituto Nacional de Enfermedades Virales Humanas, Pergamino, Buenos Aires, Argentina

### References

1. Zaidenberg M. Emergencia de dengue en la Argentina. Epidemia de dengue en Salta. Epidemiología y Vacunas;1999; 3:1-4.
2. Gandolfo F, Gonzalez H. Dengue. In: Lopez A, editor. Clínica de las Enfermedades Infecciosas y su Tratamiento. 3rd ed. Buenos Aires; 1945. p. 494-500.
3. Gaudino NM. Dengue. Revista de Sanidad Militar Argentina 1916; 15:617-27.
4. ProMed. Dengue Paraguay (12-03-00), Yellow fever (18-01-00). http/www.promedmail.org.
5. Laferté J, Pelegrino JL, Guzmán MG, González G, Vazquez S, Hermida C. Rapid diagnosis of dengue virus infection using a novel 10 µl IgM antibody capture ultramicroELISA assay (MAC UMELISA Dengue). Advances in Modern Biotechnology 1992;1:194.
6. Russel PK, Nisalak A, Sukhavachna P, Vivona S. A plaque reduction test for dengue virus neutralizing antibodies. J Immunol 1967;99:291-6.
7. Instituto Nacional de Estadísticas y Censos (INDEC). Sinopsis Estadística Argentina. Buenos Aires: INDEC; 1997.
8. Schweigmann N, Boffi R. *Aedes aegypti* y *Aedes albopictus*: Situación entomológica en la región en temas de zoonosis y enfermedades emergentes. Segundo Cong. Argent. de Zoonosis y Primer Cong. Argent. y Lationoamer. de Enf. Emerg. y Asociación Argentina de Zoonosis. Buenos Aires: Asociación Argentina de Zoonosis; 1998. p. 259-63.
9. Secretaría de Turismo de la Nación. El turismo en cifras. Años 1990-1997. Buenos Aires: the Secretaría; 1998. p. 1-9.

